# Root Cortex Provides a Venue for Gas-Space Formation and Is Essential for Plant Adaptation to Waterlogging

**DOI:** 10.3389/fpls.2019.00259

**Published:** 2019-03-29

**Authors:** Takaki Yamauchi, Fumitaka Abe, Nobuhiro Tsutsumi, Mikio Nakazono

**Affiliations:** ^1^ Japan Science and Technology Agency, PRESTO, Kawaguchi, Japan; ^2^ Graduate School of Agricultural and Life Sciences, University of Tokyo, Tokyo, Japan; ^3^ Institute of Crop Science, NARO, Tsukuba, Japan; ^4^ Graduate School of Bioagricultural Sciences, Nagoya University, Nagoya, Japan; ^5^ UWA School of Agriculture and Environment, The University of Western Australia, Crawley, WA, Australia

**Keywords:** aerenchyma, cereal crops, cortex to stele ratio, intercellular spaces, longitudinal oxygen transport, radial oxygen loss, waterlogging

## Abstract

Lysigenous aerenchyma, which develops by death and subsequent lysis of the cortical cells in roots, is essential for internal long-distance oxygen transport from shoot base to root tips of plants in waterlogged soil. Although many studies focus on the amounts of aerenchyma in roots, significance of the size of the root cortex in which aerenchyma forms has received less research attention. In the present study, we evaluated the cross-sectional area of each root tissue in adventitious roots of upland crops, wheat (*Triticum aestivum*) and maize (*Zea mays* ssp. *mays*), and the wetland crop, rice (*Oryza sativa*) under aerated or stagnant deoxygenated conditions; the latter can mimic the changes in gas composition in waterlogged soils. Our analyses revealed that the areas of whole root and cortex of the three species increased under stagnant conditions. In rice roots, cortex to stele ratio (CSR) and aerenchyma to cortex ratio (ACR), which is associated with the areas of gas spaces, were much higher than those in wheat and maize roots, suggesting that these anatomical features are essential for a high capacity for oxygen transport along roots. To test this hypothesis, rates of radial oxygen loss (ROL), which is the diffusive flux of oxygen from within a root to the external medium, from thick and thin adventitious roots of rice were measured using a cylindrical (root-sleeving) oxygen electrode, for plants with shoots in air and roots in an oxygen-free medium. As expected, the rate of ROL from thick roots, which have larger cortex and aerenchyma areas, was higher than that of thin roots. The rate of ROL was highest at the apical part of rice roots, where aerenchyma was hardly detected, but at which cuboidal cell arrangement in the cortex provides tissue porosity. We conclude that high CSR in combination with large root diameter is a feature which promotes oxygen transport from shoot base to root tips of plants. Moreover, we propose that CSR should be a useful quantitative index for the evaluation and improvement of root traits contributing to tolerance of crops to soil waterlogging.

## Introduction

Roots are mainly composed of four concentric cell layers: from the outside to inside, these cell layers are the epidermis, cortex, endodermis, and stele (Scheres et al., 2002). The stele contains xylem vessels and phloem tissue which are essential for the transport of water, inorganic ions, and organic compounds (Nissen, 1974; Petricka et al., 2012), as well as a pith in the case of monocots (Watt et al., 2008). The primary root of *Arabidopsis* (*Arabidopsis thaliana*) has a single cortex cell layer which is produced by the asymmetric cell division of cortex/endodermis initial cell (Dolan et al., 1993; Laurenzio et al., 1996). By contrast, roots of rice (*Oryza sativa*) have several cortex cell layers produced by the sequential cell divisions of the endodermal cells (Kamiya et al., 2003). Unlike *Arabidopsis*, many plant species including the family Poaceae, members of which include important crops, have several cortex cell layers (Armstrong, 1979; Justin and Armstrong, 1987).

The principal cause of damage to plants grown in waterlogged soil is inadequate supply of oxygen to submerged roots (Armstrong, 1979; Colmer and Voesenek, 2009). Therefore, internal oxygen transport from shoot base to root tips is crucial for the survival of plants under waterlogged conditions (Armstrong, 1979; Colmer, 2003b). Diffusivity of oxygen in water is approximately 10,000-fold slower than in air (Armstrong and Drew, 2002). Because plants have no active oxygen transport mechanisms, oxygen movement from shoot to submerged tissues is dominated by diffusion (Armstrong and Armstrong, 2014). Aerenchyma consists of longitudinally connected gas spaces which enhances the diffusion of oxygen within plants (Armstrong, 1979; Colmer, 2003b). Lysigenous aerenchyma in roots is formed by the creation of gas spaces as a result of death and the subsequent lysis of cortical cells (Evans, 2003). Plants in the family Poaceae, such as wheat (*Triticum aestivum*), maize (*Zea mays* ssp. *mays*), and rice form lysigenous aerenchyma in the roots (Yamauchi et al., 2018). Under aerobic conditions, roots of wheat and maize typically form small amounts of aerenchyma, but the amount of aerenchyma nevertheless increases under oxygen-deficient conditions (Abiko et al., 2012; Mano and Omori, 2013; Yamauchi et al., 2014b). Rice forms aerenchyma constitutively, and its formation further increases under oxygen-deficient conditions (Colmer et al., 2006; Shiono et al., 2011; Yamauchi et al., 2016, 2017). Oxygen within aerenchyma will be consumed by the cells in adjacent tissues and/or diffuse radially to the rhizosphere (Armstrong, 1979; Colmer, 2003b). In addition to the large amount of aerenchyma, rice roots form a barrier impermeable to radial oxygen loss (ROL), and this further enhances longitudinal oxygen diffusion from shoot base to the root tip (Armstrong, 1979; Colmer et al., 1998; Kotula et al., 2009; Shiono et al., 2011). In contrast to the many wetland species, none of the upland crops have ROL barrier in the roots, and thus its formation is an important mechanism for plants to adapt to waterlogging (Colmer, 2003b).

The amount of longitudinal oxygen diffusion along submerged organs is determined by anatomical, morphological, and physiological characteristics (Colmer, 2003b). Root thickness is a morphological feature which associates with the porosities (i.e., gas volumes) within roots (Colmer, 2003b). Root porosities under oxygen-deficient conditions are higher in the wetland species having thicker root diameters when compared with the dryland species with thinner root diameters (Visser et al., 2000a,b). From the anatomical aspects, thick root diameter is associated with large cortex and aerenchyma areas. Indeed, thicker roots of wheat seedlings that emerge under oxygen-deficient conditions have a larger aerenchyma area than thinner roots that emerge under aerobic conditions (Yamauchi et al., 2014a). On the other hand, mathematical modelings showed that large stele in which porosity is lower than in the cortex has a disadvantage in terms of oxygen diffusion within the root (Armstrong and Beckett, 1987; Armstrong et al., 1994, 2000). Indeed, the proportion of the stele area within roots is smaller in the wetland species than that in dryland species (McDonald et al., 2002). These studies suggest that not only aerenchyma, but also root thickness and ratio of cortex-to-stele within roots are essential to consider the plant adaptability to waterlogging.

The aim of this study was to understand the significance of higher CSR, which is associated with larger cortex area, for the adaptation of plants to waterlogging. Specifically, we addressed the following questions. (1) How is CSR related with plant adaptation to waterlogging? (2) Does CSR affect the amount of gas space in root? (3) What is the adaptive value of high CSR in terms of root aeration? To this end, we evaluated growth of wheat, maize, and rice seedlings under aerated or stagnant deoxygenated conditions, which can mimic the changes in gas composition in waterlogged soils. Moreover, we measured areas of stele, cortex, and aerenchyma in cross sections of adventitious roots and calculated the ratio of each tissue. To further evaluate the advantage of larger cortex to enhance internal oxygen movement within roots, we measured rates of ROL from thick and thin adventitious roots of rice seedlings by a cylindrical oxygen electrode, when in an oxygen-free root zone. We also measured elongation of the two types of adventitious roots under stagnant conditions. Based upon these results, here we discuss the adaptive consequence of high CSR to waterlogging and the potential application of CSR to improve tolerance of crops to waterlogging.

## Materials and Methods

### Plant Materials and Growth Conditions

Seeds of wheat (cv. Bobwhite), maize (inbred line B73), and rice (cv. Nipponbare) were sterilized with 0.5% (v/v) sodium hypochlorite for 30 min and then rinsed thoroughly with deionized water. Seeds of maize and wheat were germinated on filter paper in Petri dishes with deionized water. Each species was in a different growth chamber; maize was at 28°C in light [photosynthetically active radiation (PAR), 200–250 μmol m^−2^ s^−1^] for 16 h and 25°C in dark for 8 h, and wheat at 23°C under continuous light conditions (PAR, 200–250 μmol m^−2^ s^−1^), respectively. Seeds of rice were germinated in Petri dishes with deionized water in growth chamber at 28°C under dark conditions. After 2 days, maize seedlings were placed on a mesh-float with an aerated half-strength nutrient solution [28°C in light (PAR, 200–250 µmol m^−2^ s^−1^) for 16 h and 25°C in dark for 8 h]. Wheat and rice seedlings were each placed on each different mesh-floats with an aerated quarter-strength nutrient solution [23°C (wheat) or 28°C (rice) continuous light conditions; PAR, 200–250 μmol m^−2^ s^−1^]. Composition of the nutrient solution for maize was as described by Watanabe et al. (2017), and that of the nutrient solution for wheat and rice was as described by Colmer et al. (2006). After 7 days (9 days old), wheat, maize, and rice seedlings were transferred to 2-L pots (2 plants per pot, 250 mm height × 80 mm length × 110 mm width) containing aerated full-strength nutrient solution (aerated conditions) or stagnant deoxygenated solution (stagnant conditions) and grown for 7 days, and 16-day-old seedlings were used for each experiment. For maize, FeSO_4_ (final concentration of 1 μM) was added to aerated nutrient solution every day on days 9–15. For 14 days of treatment of rice seedlings, stagnant deoxygenated solution was renewed at 7 days after the start of treatment (16 days old), and 23-day-old seedlings were used for each experiment. Stagnant deoxygenated solution contained 0.1% (w/v) dissolved agar and was deoxygenated (dissolved oxygen, <0.5 mg L^−1^) prior to use by flushing with nitrogen gas (Wiengweera et al., 1997).

### Measurements of Growth and Leaf Chlorophyll Content

Wheat, maize, and rice seedlings were harvested at 7 days (16 days old) after the transfer to aerated or stagnant conditions. Lengths of shoots, longest seminal roots, and longest adventitious roots were measured using a ruler, and number of emerged leaves, adventitious roots, and tillers were counted. Chlorophyll content of leaves was measured three times at the middle part of leaves using a Soil Plant Analysis Development (SPAD) meter (SPAD-502, Konica Minolta), and the average value was used as the SPAD value of each individual. Wheat, maize, and rice seedlings were divided into shoots and roots and dried at 50°C for 7 days, and shoot and root dry weights were measured.

### Anatomical Analysis of Root Cross Sections

Root segments at 10, 20, 30, 40, and 50 mm (±2 mm) from the tips were prepared from 80- to 100-mm-long adventitious roots of wheat, maize, and rice seedlings grown under aerated or stagnant conditions for 7 days (16 days old). For the comparison between the thick and thin adventitious roots, root segments at 5, 10, 20, 30, 40, and 50 mm (±2 mm) from the tips were prepared from 110- to 130-mm-long adventitious roots of rice seedlings grown under stagnant conditions for 14 days (23 days old). Root cross sections were prepared by hand sectioning with a razor blade. Each section was photographed using an optical microscope (BX60, OLYMPUS) with a CCD camera (DP70, OLYMPUS). Areas of each root tissue were measured using ImageJ software (Ver. 1.43u, US National Institutes of Health), and the ratio (proportion) of each root tissue was calculated using the cross-sectional areas.

### Detection of Oxygen Loss From Roots by Redox Indicator Dyes

Methylene blue is a redox indicator dye which enables qualitative assessments of spatial ROL from roots (Armstrong and Armstrong, 1988). Crystal violet was applied as an alternative redox indicator dye. The reduced forms of methylene blue and crystal violet are colorless, but when they react with oxygen this results in blue and purple colors, respectively. Methylene blue and crystal violet were added to a deoxygenated solution containing 0.1% (w/v) agar at final concentration of 30 and 10 μM, respectively. Subsequently, sodium dithionite (Na_2_S_2_O_3_) was added at a final concentration of 300 μM to reduce the indicator dyes. One each of thick and thin adventitious roots (110–130 mm long) were selected from rice seedlings grown under stagnant conditions for 14 days (23 days old), and all of the other roots were trimmed off immediately before the start of staining. The shoot base with the adventitious root was immersed in a transparent acrylic pot (350 mm height × 250 mm length × 15 mm width) containing the reduced methylene blue or crystal violet solution. The shoot-root junction was 20 mm below the surface of the solution. The staining patterns of methylene blue and crystal violet on the adventitious roots were evaluated every 30 min for a period of 2 h, and photographs were taken at 2 h (methylene blue) and 1 h (crystal violet) after the start of the staining experiments.

### Measurement of Oxygen Loss From Roots by a Cylindrical Electrode

One each of thick and thin adventitious roots (110–130 mm long) were selected from rice seedlings grown under stagnant conditions for 14 days (23 days old). Oxygen loss from the adventitious roots of intact rice seedlings was measured by using a cylindrical oxygen electrode in accordance with the method of Armstrong and Wright (1975). The adventitious roots were immersed in a transparent acrylic pot (250 mm height × 80 mm length × 110 mm width) containing a deoxygenated solution of 5 mM KCl, 0.5 mM CaSO_4_, and 0.1% (w/v) dissolved agar (Colmer, 2003a), and placed in a growth chamber [28°C, light conditions; PAR, 200–250 μmol m^−2^ s^−1^]. The shoot-root junction was immersed 20 mm below the surface of the deoxygenated solution. One adventitious root was inserted through the cylindrical electrode (inner diameter: 2.25 mm; height: 5 mm), and the oxygen loss from the root surface was measured at 5, 10, 20, 30, 40, 50, and 60 mm (±2.5 mm) from the tips of adventitious roots. After the measurements, root cross sections were prepared as described above. The rates of total oxygen loss (ng min^−1^) within the range of cylindrical electrode was calculated by [−4.974 × *I*], and the rates of ROL (ng cm^−2^ min^−1^) were calculated by [−4.974 × *I*/*A*_1_] as described by Armstrong and Wright (1975), where *I* = diffusion current (μA) with the adventitious roots in the cylindrical electrode, and the *A*_1_ = surface area of adventitious roots within the electrode (m^−2^). After the calculations, the units of the rates of total oxygen loss and ROL were converted into nmol s^−1^ and nmol m^−2^ s^−1^, respectively.

### Measurement of Root Elongation

One each of thick and thin adventitious roots (10–40 mm long) were selected from rice seedlings grown under stagnant conditions for 7 days (16 days old). The selected roots were marked by threads, and their lengths were measured every day on days 16–26. Stagnant deoxygenated solution was renewed at day 16, when the measurement started.

### Statistical Analyses

Statistical differences between means were calculated using two-sample *t*-test. For multiple comparisons, data were analyzed by one-way ANOVA and *post hoc* Tukey’s test using SPSS Statistics Version 19 (IBM Software).

## Results

### Growth of Wheat, Maize, and Rice Seedlings Under Aerated and Stagnant Conditions

**Figure 1:**
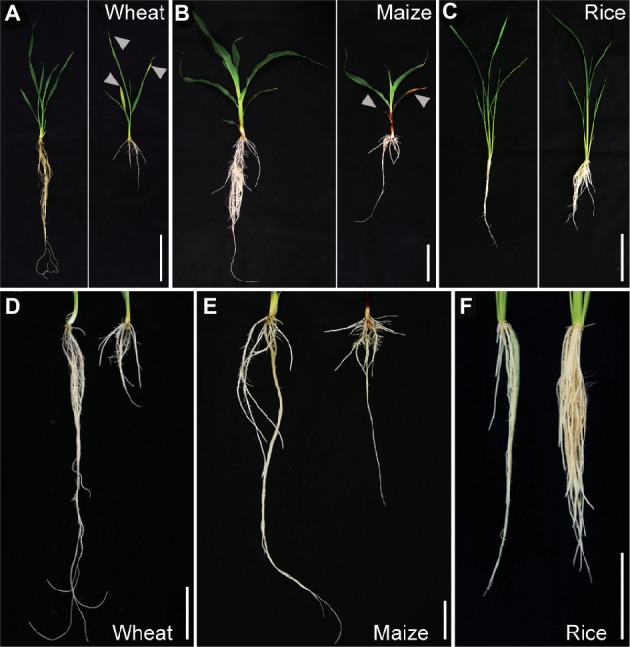
Growth of wheat, maize, and rice under aerated or stagnant conditions. Pictures of seedlings of wheat **(A)**, maize **(B)**, and rice **(C)** and roots of wheat **(D)**, maize **(E)**, and rice **(F)** under aerated conditions (left) or stagnant conditions (right). Nine-day-old wheat, maize, and rice seedlings were further grown under aerated or stagnant conditions for 7 days. Yellowing or brownish parts of leaves are indicated by arrowheads. Bars = 10 cm.

To evaluate the effect of stagnant conditions on growth of wheat, maize, and rice, 9-day-old aerobically grown seedlings were further grown under aerated or stagnant conditions for 7 days (Figures 1A–F). Stagnant conditions significantly decreased the shoot lengths of wheat and maize, and also reduced leaf and tiller numbers of maize and wheat, respectively (Table 1). Shoot dry weights of wheat and maize decreased by 11 and 13%, respectively (Table 1). In rice, stagnant conditions did not affect shoot length, leaf, and tiller numbers, and thus shoot dry weight was similar under aerated and stagnant conditions (Table 1). The chlorophyll content (SPAD value) of second and third leaves of wheat and maize significantly decreased under stagnant conditions (Table 1). In rice, the chlorophyll content of the second leaf was not affected, and that of the third leaf rather increased when plants were growing under stagnant conditions (Table 1).

**Table 1:** Growth of wheat, maize, and rice under aerated or stagnant conditions.

Conditions	Length (mm)			Number			Dry weight (mg)		SPAD	
	Shoot	Longest seminal root	Longest adventitious root	Leaf	Adventitious root	Tiller	Shoot	Root	Second leaf	Third leaf
Wheat (*Triticum aestivum*) cv. Bobwhite										
Aerated	277.7 ± 19.1	370.1 ± 32.7	155.3 ± 23.2	4.0 ± 0.0	5.7 ± 1.0	2.3 ± 0.6	193.5 ± 22.2	74.7 ± 10.0	52.6 ± 2.8	49.0 ± 3.3
Stagnant	233.5 ± 19.1^**^	n.d.	88.3 ± 10.3^**^	4.0 ± 0.0	6.5 ± 0.6^*^	1.3 ± 0.5^**^	172.1 ± 15.7^*^	22.5 ± 5.1^**^	24.3 ± 12.7^**^	37.1 ± 5.6^**^
% of control^a^	16	–	43	(0)	14	44	11	70	54	24
Maize (*Zea mays*) inbred line B73										
Aerated	353.4 ± 22.9	501.0 ± 53.9	282.9 ± 35.1	6.0 ± 0.4	8.1 ± 1.6	0.0 ± 0.0	431.4 ± 77.8	151.4 ± 26.7	43.0 ± 6.0	47.4 ± 6.1
Stagnant	303.3 ± 22.8^**^	n.d.	93.7 ± 9.5^**^	5.2 ± 0.4^**^	8.1 ± 1.1	0.0 ± 0.0	374.9 ± 35.3^*^	109.9 ± 12.4^**^	6.3 ± 4.0^**^	31.7 ± 5.4^**^
% of control^a^	14	–	67	13	(1)	–	13	27	85	33
Rice (*Oryza sativa*) cv. Nipponbare										
Aerated	309.7 ± 28.5	n.d.	168.2 ± 13.4	5.0 ± 0.0	19.7 ± 2.3	1.8 ± 0.6	122.5 ± 17.5	24.1 ± 4.0	39.2 ± 1.4	30.5 ± 3.8
Stagnant	324.7 ± 20.8	n.d.	133.7 ± 16.4^**^	5.0 ± 0.0	25.5 ± 2.5^**^	1.8 ± 0.4	126.9 ± 19.9	55.9 ± 15.4^**^	40.0 ± 2.4	41.4 ± 1.8^**^
% of control^a^	(5)	–	21	(0)	29	(0)	(4)	132	(2)	36

Nine-day-old rice, wheat, and maize seedlings were grown under aerated or stagnant conditions for 7 days, and all growth parameters were measured immediately after the treatments.

Values are means ± SD (n = 15). Significant differences between aerated and stagnant conditions at **p < 0.01 and *p < 0.05 (two-sample t-test).

Numbers within parentheses indicate that the percentage increase or decrease is not significant.

a Percentage of increase (red font) or decrease (blue font) in growth parameters under stagnant conditions in comparison with those under aerated conditions.

Root dry weights of wheat and maize respectively decreased by 70 and 27% under stagnant conditions, whereas that of rice increased by 132% (Table 1). The lengths of longest adventitious roots decreased by 43, 67, and 21% in wheat, maize, and rice under stagnant conditions, respectively (Table 1). Stagnant conditions increased the numbers of adventitious roots in wheat and rice by 14 and 29%, respectively (Table 1). By contrast, the number of adventitious roots in maize was not affected (Table 1). Seminal roots strongly contribute to the root dry weights of wheat and maize under aerated conditions, but most of those roots died during the growth under stagnant conditions (Figures 1D, E; Table 1). These results clearly show that rice is more adaptive to stagnant conditions (which mimic waterlogging) than wheat and maize.

### Size of Each Root Tissue of Wheat, Maize, and Rice Under Aerated and Stagnant Conditions

**Figure 2:**
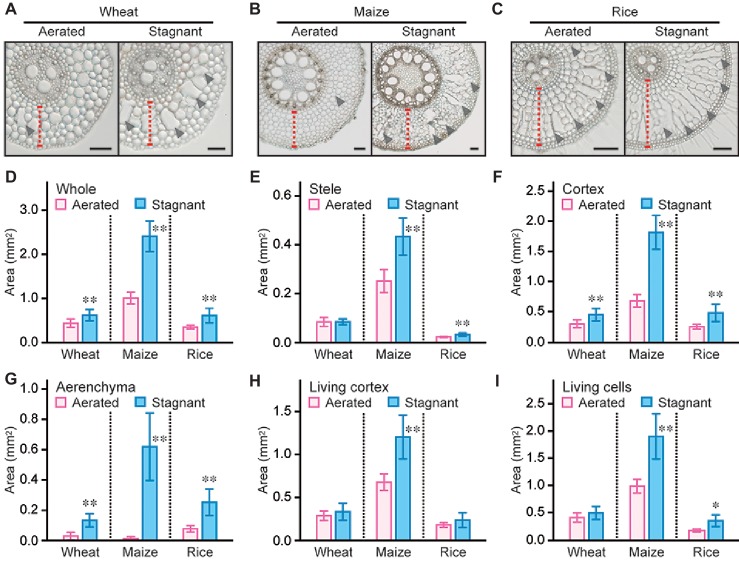
Area of each tissue in adventitious roots of wheat, maize, and rice. Cross sections at 50 mm from the tips of adventitious roots of wheat **(A)**, maize **(B)**, and rice **(C)** under aerated or stagnant conditions. Cortex cell layers and lysigenous aerenchyma are indicated by red dashed lines and black arrowheads, respectively. Bars = 100 μm. Areas of whole root **(D)**, stele **(E)**, cortex **(F)**, aerenchyma **(G)**, living cortex (cortex minus aerenchyma) **(H)**, and living cells (whole root minus aerenchyma) **(I)** at 50 mm from the tips of adventitious roots of wheat, maize, and rice seedlings under aerated or stagnant conditions. Nine-day-old wheat, maize, and rice seedlings were further grown under aerated or stagnant conditions for 7 days. Significant differences between aerated and stagnant conditions at *p* < 0.01 and *p* < 0.05 (two-sample *t*-test) are denoted by ∗∗ and ∗, respectively. Values are means ± SD (*n* = 9).

Root cross sections at 10, 20, 30, 40, and 50 mm from the tips of 80- to 100-mm-long adventitious roots were prepared from wheat, maize, and rice seedlings grown under aerated or stagnant conditions, and the size of each root tissue was measured (at 50 mm; Figures 2A–C). Areas of whole root, stele, and cortex at 50 mm from the tips of wheat, maize, and rice roots, except for stele area of wheat roots, significantly increased under stagnant conditions (Figures 2D–F). Stele areas in maize and rice roots increased by 72 and 40%, respectively (Figure 2E). In wheat, maize, and rice, areas of cortex respectively increased by 49, 164, and 88% under stagnant conditions (Figure 2F). These results indicate that the increases of the cortex areas are greater than those of stele areas under stagnant conditions. Similar results were obtained at 10, 20, 30, and 40 mm from the tips of adventitious roots of wheat, maize, and rice seedlings (Supplementary Figures S1A–C, S2A–C, S3A–C).

Although aerenchyma formation was minor in wheat and maize roots under aerated conditions, aerenchyma formation gradually increased toward basal parts of the roots under stagnant conditions (Supplementary Figures S1D, S2D). In rice roots, aerenchyma formation was first detected at 20 mm from the tips both under aerated and stagnant conditions, and its formation gradually increased toward basal parts of the roots (Supplementary Figure S3D). Areas of aerenchyma at 50 mm from the root tips significantly increased in wheat, maize, and rice roots under stagnant conditions (Figure 2G).

Because the area of whole roots in maize strongly increased under stagnant conditions (Figure 2D), areas of living cortex (cortex minus aerenchyma; 77%) and living root cells (whole roots minus aerenchyma; 92%) also strongly increased (Figures 2H,I). In wheat roots, areas of living cortex and living root cells were comparable between aerated and stagnant conditions (Figures 2H, I). In rice roots, areas of living cortex were comparable between aerated and stagnant conditions, and the area of living root cells increased by 32% under stagnant conditions (Figures 2H, I). Areas of living cortex gradually decreased toward basal parts of the roots in maize and rice, but not in wheat, under stagnant conditions (Supplementary Figures S1E, S2E, S3E). Area of living root cells in rice also gradually decreased toward the basal part of the roots, whereas those of living root cells did not significantly change along wheat and maize roots (Supplementary Figures S1F, S2F, S3F).

### Ratio of Each Root Tissue Size of Wheat, Maize, and Rice Under Aerated and Stagnant Conditions

**Figure 3:**
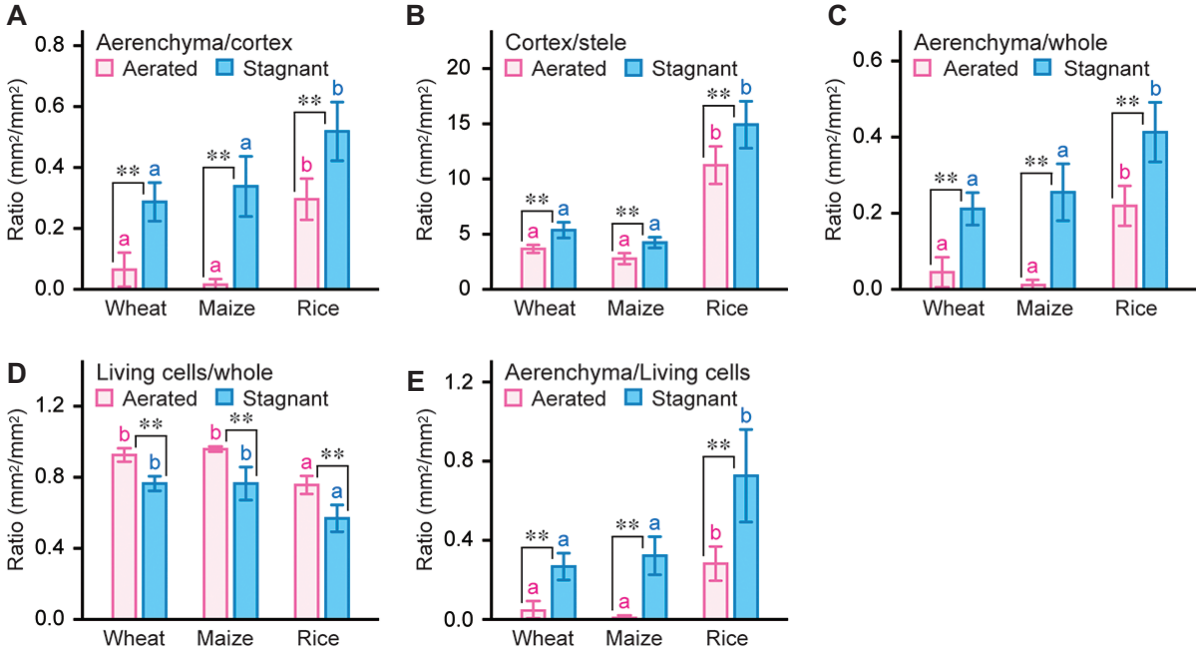
Ratio of each tissue size in adventitious roots of wheat, maize, and rice. Ratio of aerenchyma to cortex **(A)**, cortex to stele **(B)**, aerenchyma to whole root **(C)**, living cells to whole root **(D)**, and aerenchyma to living cells **(E)** at 50 mm from the tips of adventitious roots of wheat, maize, and rice under aerated or stagnant conditions. Nine-day-old wheat, maize, and rice seedlings were further grown under aerated or stagnant conditions for 7 days. Significant differences between aerated and stagnant conditions at p < 0.01 (two-sample *t*-test) are denoted by ∗∗. Different lowercase letters denote significant differences among different species (*p* < 0.05, one-way ANOVA and then Tukey’s test for multiple comparisons). Values are means ± SD (*n* = 9).

To compare the root anatomical features of wheat, maize, and rice, the ratio of each root tissue was calculated (Figure 3; Supplementary Figures S4–S6). Stagnant conditions significantly increased aerenchyma to cortex ratio (ACR) and cortex to stele ratio (CSR) at 50 mm from the tips of adventitious roots in wheat, maize, and rice (Figures 3A,B). ACR and CSR in rice roots were much higher than those in wheat and maize roots both under aerated and stagnant conditions (Figures 3A, B). ACR in rice roots under stagnant conditions was respectively 1.8-fold and 1.5-fold higher than that in wheat and maize roots (Figure 3A), and CSR in rice roots under stagnant conditions was respectively 2.8-fold and 3.5-fold higher than that in wheat and maize roots (Figure 3B). These results indicate that rice roots have more aerenchyma within cortex when compared with wheat and maize roots, and suggest that higher CSR in rice roots further amplifies the ratio of aerenchyma within whole root. Indeed, aerenchyma to whole root ratio in rice roots under stagnant conditions was respectively 2.0-fold and 1.6-fold higher than that in wheat and maize roots (Figure 3C), and the proportion of difference between rice roots and wheat or maize roots was larger than ACR (Figures3A, C). Moreover, ratio of living cells to whole root in rice roots under stagnant conditions was 1.3-fold lower than that in wheat and maize roots under stagnant conditions (Figure 3D), and ratio of aerenchyma to living cells in rice roots was respectively 2.7-fold and 2.2-fold higher than that in wheat and maize roots under stagnant conditions (Figure 3E). These results indicate that rice has larger area of aerenchyma and smaller area of living cells within roots when compared with wheat and maize. In other words, rice roots suppress oxygen demands of living cells and enhance oxygen diffusion rates through aerenchyma to adapt to waterlogging.

### Anatomy of Thick and Thin Rice Roots Under Stagnant Conditions

**Figure 4:**
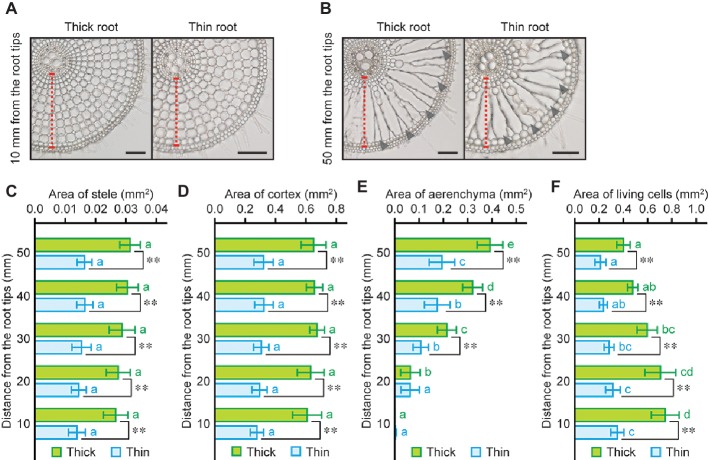
Size of each tissue in thick and thin adventitious roots of rice. Cross sections at 10 mm **(A)** and 50 mm **(B)** from the tips of thick and thin adventitious roots of rice under stagnant conditions. Cortex cell layers and lysigenous aerenchyma are indicated by red dashed lines and black arrowheads, respectively. Bars = 100 μm. Area of stele **(C)**, cortex **(D)**, aerenchyma **(E)**, and living cells **(F)** at 10, 20, 30, 40, and 50 mm from the tips of thick and thin adventitious roots of rice seedlings under stagnant conditions. Nine-day-old rice seedlings were further grown under stagnant conditions for 14 days. Significant differences between thick and thin roots at p < 0.01 (two-sample *t*-test) are denoted by ∗∗. Different lowercase letters denote significant differences among different positions of roots (*p* < 0.05, one-way ANOVA and then Tukey’s test for multiple comparisons). Values are means ± SD (*n* = 8).

Analysis of root anatomical features in wheat, maize, and rice revealed that the ratio of cortex area within roots in rice is higher than that in wheat and maize roots (Figure 3B). To demonstrate the advantages of high CSR in rice roots for oxygen transport from shoot to root, one each of thick and thin adventitious roots (110- to 130-mm long) were selected from each rice seedling grown under stagnant conditions for 14 days, and anatomical features of those roots were evaluated (Figures 4A, B). Whole root areas at 10, 20, 30, 40, and 50 mm from the tips of thick roots were 1.9- to 2.1-fold larger than those of thin roots (Supplementary Figure S7A). Although stele and cortex areas of thick roots were respectively 1.8- to 1.9-fold and 2.0- to 2.2-fold larger than those of thin roots (Figures 4C, D), CSR was not significantly different between the two types of roots (Supplementary Figure S7B). Areas of aerenchyma at 10 and 20 mm of thick and thin roots were comparable with each other, whereas those at 30–50 mm were 1.8- to 2.0-fold larger in thick roots (Figure 4E). Areas of living cells of thick roots were 1.9- to 2.2-fold larger than those of thin roots (Figure 4F). Interestingly, the longitudinal gradients of areas of aerenchyma and living cells were more prominent in thick roots than those in thin roots (Figures 4E, F). Aerenchyma to whole root ratio, living cells to whole root ratio, and aerenchyma to living cells ratio were not significantly different between thick and thin roots (Supplementary Figures S7C–E). These results suggest that thick roots have larger oxygen demands at the apical part due to having more living cells, and that thick roots have larger amounts of aerenchyma at the basal part when compared with thin roots. Taken together, these features could synergistically enhance oxygen diffusion from shoot base to the tips of thick roots.

### Oxygen Loss From Thick and Thin Rice Roots Under Stagnant Conditions

**Figure 5:**
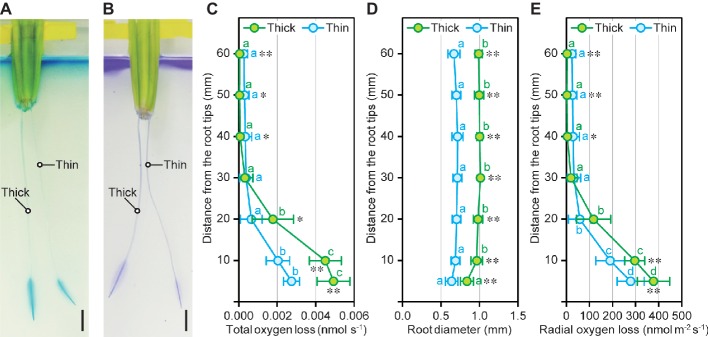
Oxygen movement in thick and thin adventitious roots of rice. Methylene blue staining **(A)** and crystal violet staining **(B)** of thick and thin adventitious roots of rice seedlings under stagnant conditions. Bars = 10 mm. Total oxygen loss from adventitious roots **(C)**, adventitious root diameter **(D)**, and radial oxygen loss (ROL) from adventitious roots **(E)** at 5, 10, 20, 30, 40, 50, and 60 mm from the tips of thick and thin adventitious roots of rice seedlings under stagnant conditions. Nine-day-old rice seedlings were further grown under stagnant conditions for 14 days. Significant differences between thick and thin roots at *p* < 0.01 and *p* < 0.05 (two-sample *t*-test) are denoted by ∗∗ and ∗, respectively. Different lowercase letters denote significant differences among different positions of roots (*p* < 0.05, one-way ANOVA and then Tukey’s test for multiple comparisons). Values are means ± SD (*n* = 8).

To evaluate the contribution of cortex to the oxygen transport from shoot base to the root tips, oxygen loss from the thick and thin adventitious roots of rice seedlings were detected by redox indicator dyes, methylene blue, and crystal violet, under the deoxygenated solution. We found that thick roots with larger cortex and aerenchyma areas showed more dense and broad staining patterns (Figures 5A, B). Subsequently, rates of oxygen loss within a cylindrical oxygen electrode (total oxygen loss) from thick and thin adventitious roots were measured at 5, 10, 20, 30, 40, 50, and 60 mm (±2.5 mm) in deoxygenated solution (Figure 5C). The total oxygen loss was hardly detectable at 30–60 mm from the tips of thick and thin roots, indicating that the both types of roots had formed a strong barrier to ROL in our experimental conditions (Figure 5C). Although the patterns of total oxygen loss along roots were similar between both types of roots, the rates of total oxygen loss at 5 and 10 mm of thick roots were 1.8- and 2.2-fold higher than those in thin roots, respectively (Figure 5C). Root diameters of thick roots at 5–60 mm from the tips were 1.3- to 1.5-fold higher than those in thin roots (Figure 5D). Nevertheless, the rates of ROL (total oxygen loss/root surface area) at 5 and 10 mm of thick roots were 1.4- and 1.6-fold higher than those of thin roots (Figure 5E). These results indicate that large cortex and aerenchyma areas strongly contribute to the efficient oxygen transport within rice roots.

### Intercellular Space of Thick and Thin Rice Roots Under Stagnant Conditions

**Figure 6:**
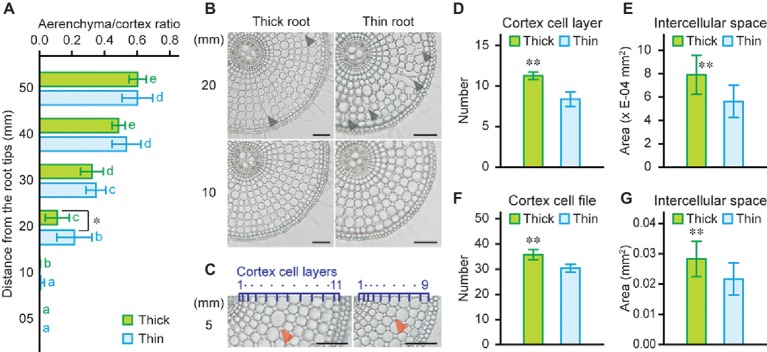
Intercellular spaces of thick and thin adventitious roots of rice. **(A)** Ratio of aerenchyma to cortex at 5, 10, 20, 30, 40, and 50 mm from the tips of thick and thin adventitious roots of rice under stagnant conditions. **(B)** Cross-sections at 10 and 20 mm from the tips of thick and thin adventitious roots under stagnant conditions. Lysigenous aerenchyma is indicated by black arrowheads. Bars = 100 μm. **(C)** Cross-sections at 5 mm from the tips of thick and thin adventitious roots under stagnant conditions. Cortex cell layers are indicated by dark blue numbers and dots. Intercellular spaces are indicated by red arrowheads. Bars = 100 μm. Number of cortex cell layers **(D)**, area of intercellular spaces (per two cortical cell files) **(E)**, number of cortex cell files at the middle part of cortex cell layers **(F)**, and area of total intercellular spaces **(G)** at 5 mm from the tips of thick and thin adventitious roots under stagnant conditions. Nine-day-old rice seedlings were further grown under stagnant conditions for 14 days. **(A,D–G)** Significant differences between thick and thin adventitious roots at *p* < 0.01 and *p* < 0.05 (two-sample *t-*test) are denoted by ∗∗ and ∗, respectively. **(A)** Different lowercase letters denote significant differences among different positions of roots (*p* < 0.05, one-way ANOVA and then Tukey’s test for multiple comparisons). Values are means ± SD (*n* = 8).

High rates of oxygen loss were detected at 5 mm from the tips of thick and thin adventitious roots of rice seedlings (Figure 5E), even though aerenchyma formation was hardly detectable (at 10 mm; see Figure 4E). Although thick roots have larger cortex area (Figure 4D), areas of aerenchyma at 10 and 20 mm of thick roots were comparable with those of thin roots (Figure 4E). Moreover, ACR at 20 mm was smaller in thick roots than that in thin roots (Figures 6A, B). To further evaluate the contribution of large cortex area to the efficient oxygen transport within roots, areas of intercellular spaces at 5 mm of thick and thin roots were measured. Because number of cortex cell layers of thick roots was larger than that in thin roots (Figures 6C, D), area of intercellular spaces per two cortical cell files was significantly larger in thick roots (1.4-fold; Figure 6E). Moreover, thick roots have more cortical cell files (Figure 6F), and thus they have greater total intercellular spaces in the cortex than those in thin roots (1.7-fold; Figure 6G). Interestingly, area of intercellular spaces at 5 mm of thick roots was almost equal to half of aerenchyma area at 20 mm of thick roots (Figures 4E, 6G). These results indicate that larger cortex contributes to form larger gas spaces, which are comprised of not only aerenchyma but also intercellular spaces, and thus, it is essential for efficient internal oxygen transport from shoot base to the root tips of rice.

### Elongation of Thick and Thin Rice Roots Under Stagnant Conditions

**Figure 7:**
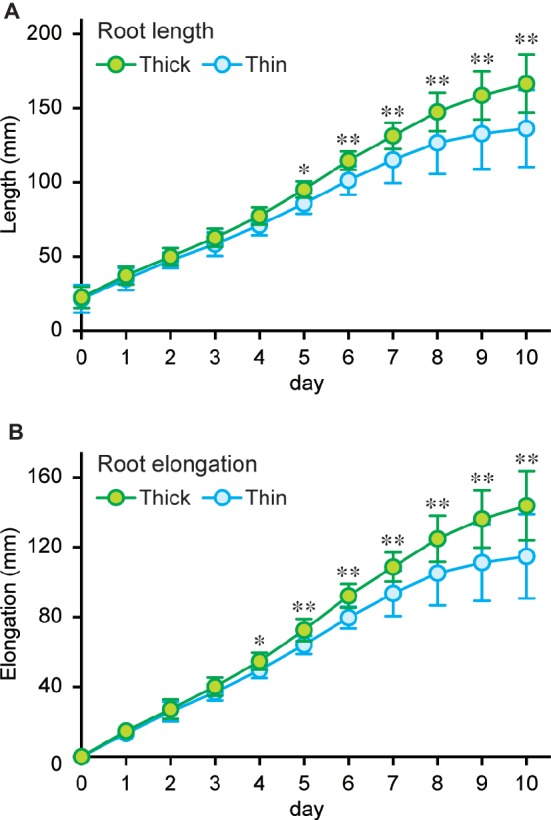
Elongation of thick and thin adventitious roots of rice. Lengths **(A)** and elongation **(B)** of thick and thin adventitious roots of rice under stagnant conditions. Nine-day-old rice seedlings were further grown under stagnant conditions for 7 days, and lengths of thick and thin adventitious roots were measured every day on days 16–26 under stagnant conditions. Significant differences between thick and thin adventitious roots at *p* < 0.01 and *p* < 0.05 (two-sample *t-*test) are denoted by ∗∗ and ∗, respectively. Values are means ± SD (*n* = 12).

To further evaluate the advantage of larger gas spaces in thick rice roots on the growth under stagnant conditions, lengths of thick and thin adventitious roots were measured every day up to 10 days (on days 16–26) under stagnant conditions (Figure 7A). Root lengths were comparable between thick and thin roots until 4 days after the start of treatment (Figure 7A). Elongation rate of thin roots became slower than that of thick roots at day 4, and it almost stopped on days 9–10 (Figure 7B). By contrast, the elongation rate of thick roots did not change until day 10 (Figure 7B), and thus differences in lengths of thick and thin roots gradually increased between days 5 and 10 (Figure 7A). These results further support that thick roots with larger cortex and gas spaces are better adapted to waterlogged conditions than thin roots.

## Discussion

Growth of rice under stagnant conditions, which mimic the changes in gas composition in waterlogged soil, was greater than for upland crops, wheat and maize (Figure 1; Table 1). Roots of rice, which is generally cultivated in waterlogged paddy fields, have higher ratio of aerenchyma within roots (i.e., ACR) and higher ratio of cortex to stele (i.e., CSR) than those of wheat and maize both under aerated and stagnant conditions (Figures 3A, B). Because the cortex is the tissue in which aerenchyma formation occurs, large CSR in rice roots amplifies the aerenchyma to whole root ratio (Figure 3C), and this also results in rice roots maintaining a smaller amount of living cells (Figure 3D). On the other hand, whole root areas increased in wheat, maize, and rice under stagnant conditions (Figure 2D), and this led to the increases in cortex areas of the three species (Figure 2F). These results suggest that increase in root diameter is a common trait for wetland and upland species in response to waterlogging. While aerenchyma is essential for the efficient internal oxygen transport within roots (Colmer, 2003b; Yamauchi et al., 2018), the cortical cell death (i.e., aerenchyma formation) also contributes to reduce the respiratory costs of roots under oxygen-deficient conditions (Drew, 1997). Indeed, larger CSR in rice roots (Figure 3B), which was associated with higher aerenchyma to whole root ratio and less living cells to whole root ratio (Figures 3C,D), was associated with the better growth of rice under stagnant conditions when compared with wheat and maize having smaller CSRs in the roots (Figure 3B; Table 1). Stele contains xylem vessels and which are essential for water and nutrient transports within roots (Petricka et al., 2012). In maize and barley (*Hordeum vulgare*), anoxia (no oxygen) or hypoxia (low oxygen) in the stelar cells severely restricted the loading of essential ions into the xylem (Gibbs et al., 1998; Colmer and Greenway, 2011; Kotula et al., 2015). Because the proportion of the stele area within roots is smaller in the wetland species than that in dryland species (McDonald et al., 2002), smaller stele which is associated with larger cortex and aerenchyma areas, could be more adaptive to waterlogged conditions.

Shoot and root dry weights of maize seedlings were much greater than those of wheat and rice seedlings both under aerated and stagnant conditions (Table 1), and the stele area of maize roots was also much larger than those of wheat and rice roots (Figure 2E). A study of 19 grass species showed that plant height at maturity stages correlated positively with the root cross-sectional areas of stele (Pearson correlation coefficient, *r* = 0.52, *p* < 0.05) and total xylems (*r* = 0.63, *p* < 0.01; Wahl and Ryser, 2000), suggesting that smaller capacities of water and nutrient transports (i.e., smaller stele and xylem vessels) is a disadvantage for the growth of plants. One way to resolve this problem would be to increase the number of roots to compensate less effective water and nutrient transports caused by the smaller stele and xylem sizes. Indeed, adventitious root number of the wetland species *Rumex palustris* is much larger than that of the non-wetland species *Rumex thyrsiflorus* both under aerated and stagnant conditions (Visser et al., 1995, 1996). In our experimental conditions, CSR in rice roots was much higher than that in wheat and maize roots, and rice also has a much larger number of roots than wheat and maize (Figure 3B; Table 1). Interestingly, both CSR and root numbers in wheat and rice significantly increased under stagnant conditions (Figure 3B; Table 1). By contrast, root number in maize was not significantly different between aerated and stagnant conditions (Table 1). Previous study showed that root number of another maize inbred line Mi29 is comparable between under aerated and stagnant conditions, whereas the soil waterlogging significantly increases root number when compared with that under drained soil conditions (Abiko et al., 2012). Although further studies are required to demonstrate the correlation between CSR and root number of plants under waterlogging, having high cortex ratio is essential to express the efficient oxygen transport from shoot base to root tips. On the other hand, rice (and other wetland species) may require a large number of roots to compensate for the less efficient water and nutrient uptakes due to the smaller stele ratio than upland species (Figure 3B).

From the results of anatomical analyses along the adventitious roots, we found that the patterns of areas of living cells along roots were different among wheat, maize, and rice (Supplementary Figures S1F, S2F, S3F). In wheat and maize under stagnant conditions, the areas of living cells at apical parts of the roots were not much changed when compared with those at basal parts of the roots (Supplementary Figures S1F, S2F), even though areas of aerenchyma gradually increased toward basal part of the roots (Supplementary Figures S1D, S2D). By contrast, the areas of living cells at apical part of rice roots was much larger than those at basal part of the roots (Supplementary Figure S3F), and the areas of aerenchyma and living cells gradually increased and decreased toward basal part of the roots, respectively (Supplementary Figures S3D,F). Longitudinal oxygen gradient along the root axis is essential for the oxygen diffusion longitudinally from shoot base to root tip (Colmer, 2003b). Oxygen within gas spaces in roots not only diffuses but also is consumed by the root cells, and thus, the death of cells in the root cortex reduces not only the physical resistance of oxygen diffusion but also the respiratory consumption within roots. (Armstrong, 1979; Drew, 1997; Colmer, 2003b). Taking these into account, longitudinal gradient of respiratory activity along roots is particularly important for the efficient oxygen transport from shoot base to root tips. In rice, basal part of the roots has more aerenchyma and less living cells than apical part of the roots (Supplementary Figures S3D,F). If the apical part of rice roots has higher oxygen demands due to larger respiratory activity of living cells, rice roots may have more prominent longitudinal oxygen gradient than wheat and maize roots, although the steepness of the gradient is also influenced by the resistance along the path.

Many wetland species, including rice, form a barrier to ROL at basal parts of the roots, and this further enhances longitudinal oxygen diffusion from shoot base to the root tips (Armstrong, 1979; Colmer, 2003b). Therefore, a barrier to ROL is one of the most important mechanisms for the roots of wetland species to adapt to waterlogging (Colmer 2003b). Profiles of the rates of ROL along thick and thin adventitious roots of rice revealed that thick roots can transport greater amounts of oxygen from shoot base to the root tips than thin roots, even though both types of roots form a barrier to ROL under stagnant conditions (Figure 5C). The rate of ROL, which is expressed on a surface area basis of root to derive the rate, in thick roots was still higher than that in the thin roots (Figure 5E), thereby demonstrating that thick roots have an ability to sustain greater internal oxygen movement than thin roots. The longitudinal gradients of the areas of aerenchyma and living cells along the roots were more prominent in thick roots than those in thin roots (Figures 4E, F), suggesting that the respiratory activity at the apical part of the roots, which results in an oxygen concentration gradient that is the driving force of oxygen diffusion (Colmer, 2003b), is higher in the thick roots than that in the thin roots, and that respiratory consumption at the basal part of roots, which in turn reduces longitudinal oxygen diffusion (Colmer, 2003b), is relatively low in thick roots. Indeed, thick roots elongated more than thin roots under stagnant conditions (Figures 7A, B), further indicating that large cortex and aerenchyma areas within thick roots contribute to efficient oxygen transport from shoot base to the root tips. Although rates of oxygen loss from roots to the external medium depend not only on the amounts of gas spaces but also on root respiration and the tightness of barriers to ROL (Colmer, 2003b), the total flux of oxygen from just behind the root tip was substantially greater for the thicker than the thinner roots (Figure 5C), which supports that these thicker roots have more internal oxygen movement. Further analysis is needed to reveal the entire pictures of all resistances and respiratory consumption along the diffusion path for internal aeration in thick and thin adventitious roots of rice, and also for the roots of wheat and maize.

Interestingly, ROL at the apical part of rice roots, where little aerenchyma formation was detected (Figure 4E), was very high under stagnant conditions (Figure 5E). Further anatomical analysis revealed that intercellular spaces in the cortex strongly contribute to constitute gas spaces in rice roots (Figure 6G), as noted also by Armstrong (1971). Moreover, area of intercellular spaces was larger in thick roots having much cortex cell layers and the cell files than that in thin roots (Figure 6G). Arrangement of the cortical cells can be divided into two types, one is cuboidal and another is hexagonal packing (Colmer, 2003b). The root porosity was much higher at the apical parts of roots with cuboidal packing of cortex cells than those with hexagonal packing of cortical cells (Armstrong, 1971; Justin and Armstrong, 1987). Interestingly, rice roots have cortex cells with cuboidal packing and wheat roots have those with hexagonal packing, and maize roots have cortex cells with mixtures of both types (Figures 2A–C; Supplementary Figures S8A–C). These results suggest that the different arrangements of cortical cells among wheat, maize, and rice roots are also associated with their adaptabilities to waterlogging. It should be noted that the amount of intercellular spaces in cortex is amplified in roots with high CSR as is the case with the amount of aerenchyma in cortex.

From the detailed anatomical analyses and ROL measurements, we suggest that high CSR with large root diameter contributes to the efficient oxygen transport from shoot base to root tips of plants, and thus, it is essential for plant adaptation to waterlogging. Soil waterlogging negatively impacts agricultural yields of upland crops all over the world (Bailey-Serres et al., 2012; Pucciariello et al., 2014; Voesenek and Bailey-Serres, 2015; Herzog et al., 2016), so that the development of waterlogging-tolerant crops by introgression of quantitative trait loci (QTL) associated with root aeration into elite cultivars is urgent (Yamauchi et al., 2018). QTLs for root aerenchyma formation in cereal crops were identified using mapping populations of barley (Zhang et al., 2016) and hybridization of a wetland wild relative *Zea nicaraguensis* with a maize inbred line (Mano et al., 2016). Here, we showed that CSR is a reliable quantitative index, which likely contributes to waterlogging tolerance and is available for consideration in future QTL analysis of cereal crops. Moreover, CSR might synergistically enhance the root aeration with aerenchyma formation. It is attractive to identify major QTL for CSR using mapping populations of cereal crops and to improve waterlogging tolerance of cereal crops in the future.

## Data Availability

All datasets generated for this study are included in the manuscript and/or the Supplementary Files.

## Author Contributions

TY designed the research plans, performed the experiments, and wrote the article. MN provided important advice on the research plans, and read the article and gave suggestions. FA and NT provided advice on the research plans, and read the article and gave suggestions.

### Conflict of Interest Statement

The authors declare that the research was conducted in the absence of any commercial or financial relationships that could be construed as a potential conflict of interest.
