# Supplementary material for: Root Cortex Provides a Venue for Gas-Space Formation and Is Essential for Plant Adaptation to Waterlogging
Source: Front Plant Sci. 2019 Mar 29;10:259. doi: 10.3389/fpls.2019.00259 (PMC6465681; doi:10.3389/fpls.2019.00259)
Supplement: Supplementary file 1 [file Data_Sheet_1.PDF]

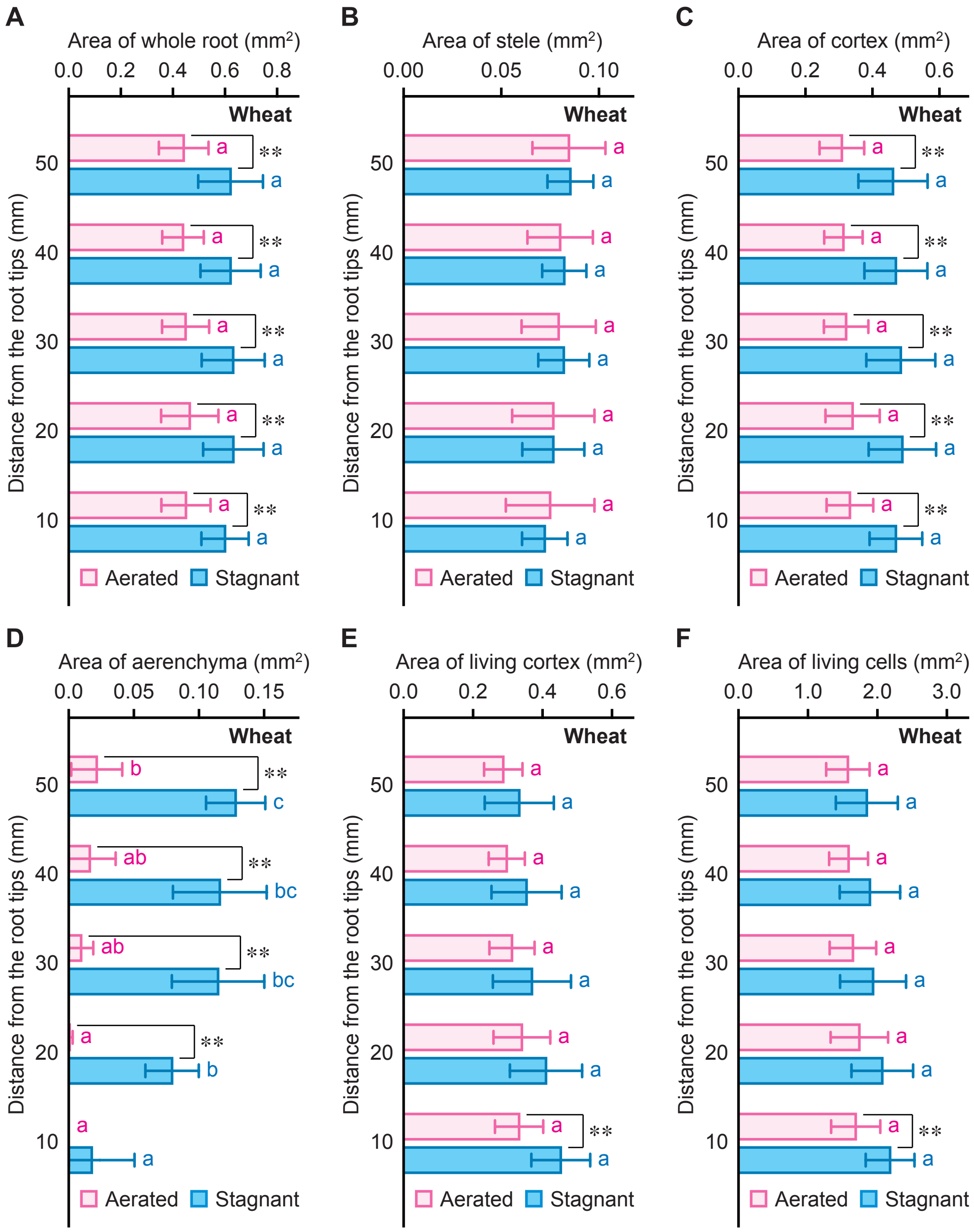

FIGURE S1 | Area of each tissue in adventitious roots of wheat. Areas of whole root **(A)**, stele **(B)**, cortex **(C)**, aerenchyma **(D)**, living cortex (cortex minus aerenchyma) **(E)**, and living cells (whole root minus aerenchyma) **(F)** at 10, 20, 30, 40, and 50 mm from the tips of adventitious roots of wheat under aerated or stagnant conditions. Nine-day-old wheat seedlings were further grown under aerated or stagnant conditions for 7 days. Significant differences between under aerated and stagnant conditions at $P < 0.01$ (two-sample *t* test) are denoted by **. Different lower-case letters denote significant differences among different positions of roots ($P < 0.05$, one-way ANOVA and then Tukey' s test for multiple comparisons). Values are means ± SD (n = 9).

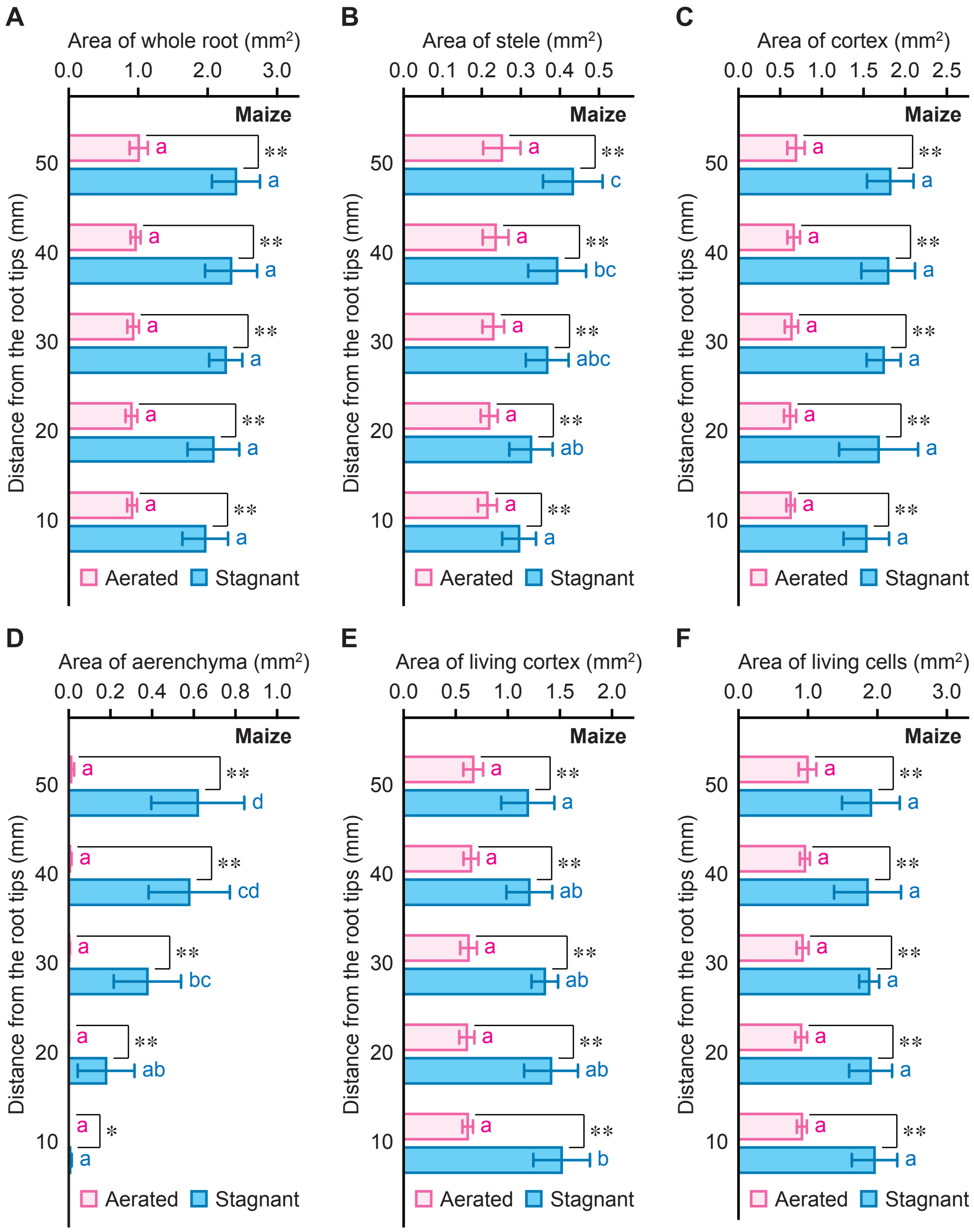

FIGURE S2 | Area of each tissue in adventitious roots of maize. Areas of whole root **(A)**, stele **(B)**, cortex **(C)**, aerenchyma **(D)**, living cortex (cortex minus aerenchyma) **(E)**, and living cells (whole minus aerenchyma) **(F)** at 10, 20, 30, 40, and 50 mm from the tips of adventitious roots of maize under aerated or stagnant conditions. Nine-day-old maize seedlings were further grown under aerated or stagnant conditions for 7 days. Significant differences between under aerated and stagnant conditions at $P < 0.01$ and $P < 0.05$ (two-sample *t* test) are denoted by ** and *, respectively. Different lower-case letters denote significant differences among different positions of roots ($P < 0.05$, one-way ANOVA and then Tukey' s test for multiple comparisons). Values are means ± SD (n = 9).

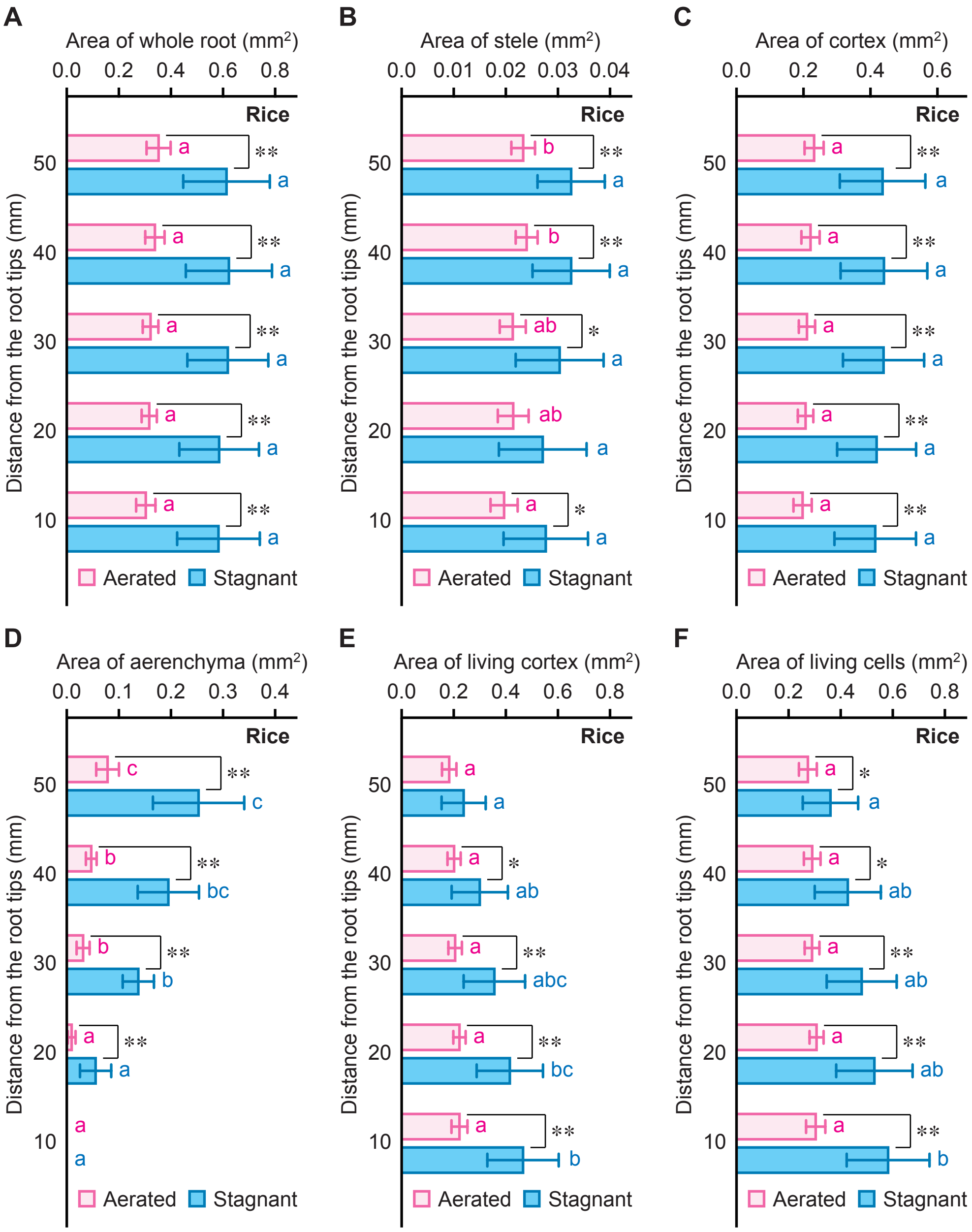

FIGURE S3 | Area of each tissue in adventitious roots of rice. Areas of whole root **(A)**, stele **(B)**, cortex **(C)**, aerenchyma **(D)**, living cortex (cortex minus aerenchyma) **(E)**, and living cells (whole minus aerenchyma) **(F)** at 10, 20, 30, 40, and 50 mm from the tips of adventitious roots of rice under aerated or stagnant conditions. Nine-day-old rice seedlings were further grown under aerated or stagnant conditions for 7 days. Significant differences between under aerated and stagnant conditions at P < 0.01 and P < 0.05 (two-sample t test) are denoted by ** and *, respectively. Different lower-case letters denote significant differences among different positions of roots (P < 0.05, one-way ANOVA and then Tukey' s test for multiple comparisons). Values are means ± SD (n = 9).

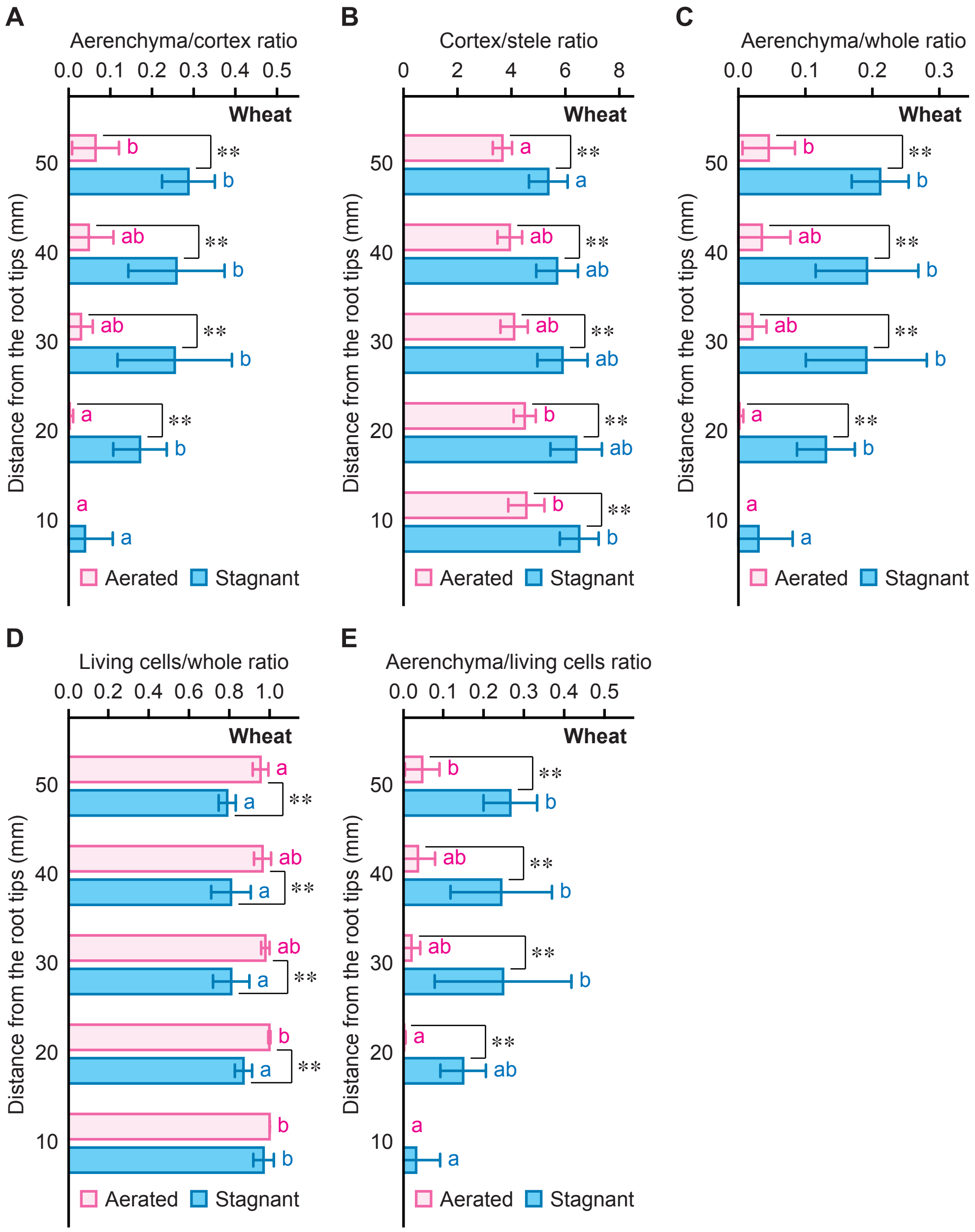

FIGURE S4 | Ratio of each tissue size in adventitious roots of wheat. Ratio of aerenchyma to cortex **(A)**, cortex to stele **(B)**, aerenchyma to whole root **(C)**, living cells to whole root **(D)**, and aerenchyma to living cells **(E)** at 10, 20, 30, 40, and 50 mm from the tips of adventitious roots of wheat under aerated or stagnant conditions. Nine-day-old wheat seedlings were further grown under aerated or stagnant conditions for 7 days. Significant differences between under aerated and stagnant conditions at $P < 0.01$ (two-sample t test) are denoted by **. Different lower-case letters denote significant differences among different positions of roots ($P < 0.05$, one-way ANOVA and then Tukey' s test for multiple comparisons). Values are means ± SD (n = 9).

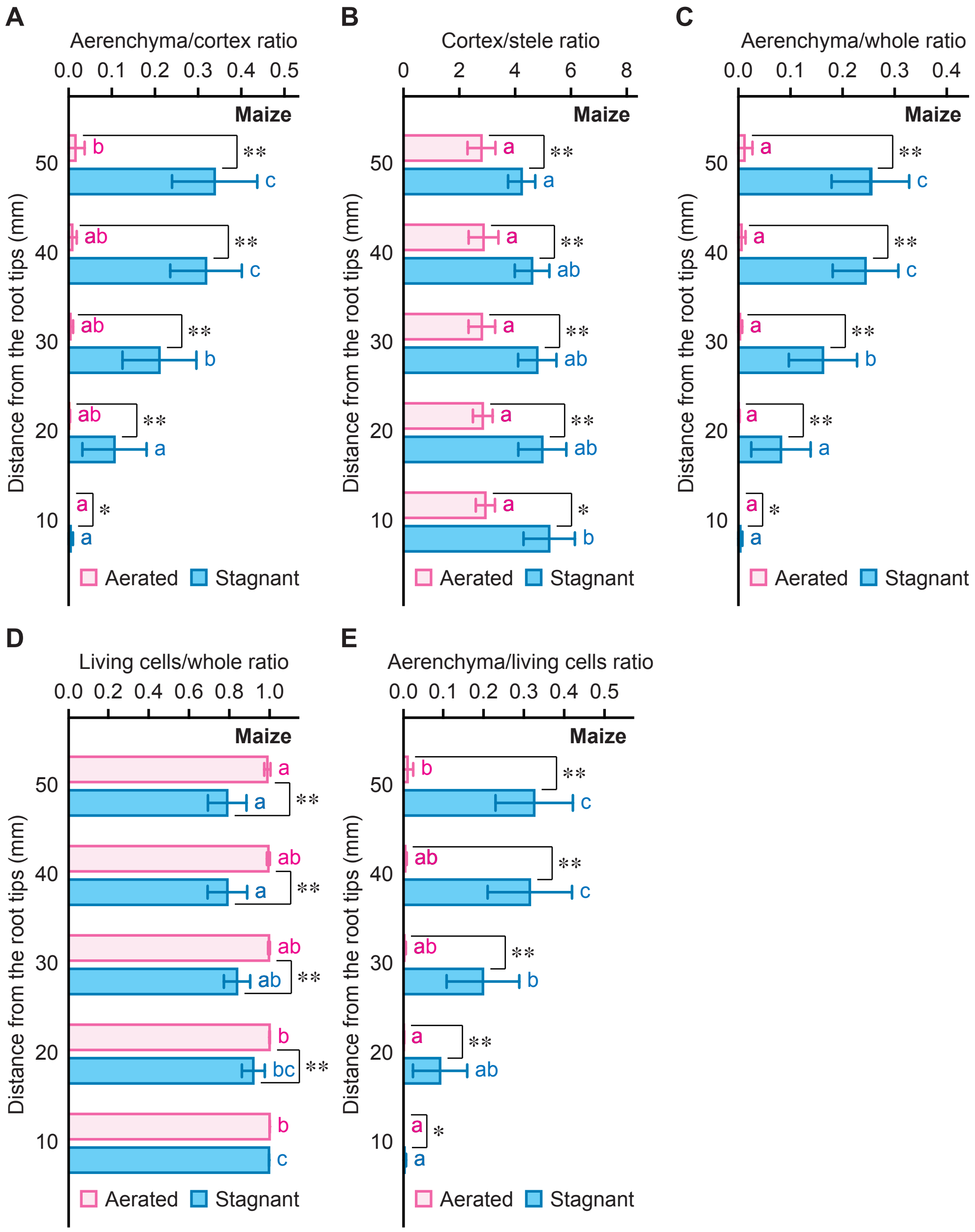

FIGURE S5 | Ratio of each tissue size in adventitious roots of maize. Ratio of aerenchyma to cortex **(A)**, cortex to stele **(B)**, aerenchyma to whole root **(C)**, living cells to whole root **(D)**, and aerenchyma to living cells **(E)** at 10, 20, 30, 40, and 50 mm from the tips of adventitious roots of maize under aerated or stagnant conditions. Nine-day-old maize seedlings were further grown under aerated or stagnant conditions for 7 days. Significant differences between under aerated and stagnant conditions at $P < 0.01$ and $P < 0.05$ (two-sample t test) are denoted by ** and *, respectively. Different lower-case letters denote significant differences among different positions of roots ($P < 0.05$, one-way ANOVA and then Tukey' s test for multiple comparisons). Values are means ± SD (n = 9).

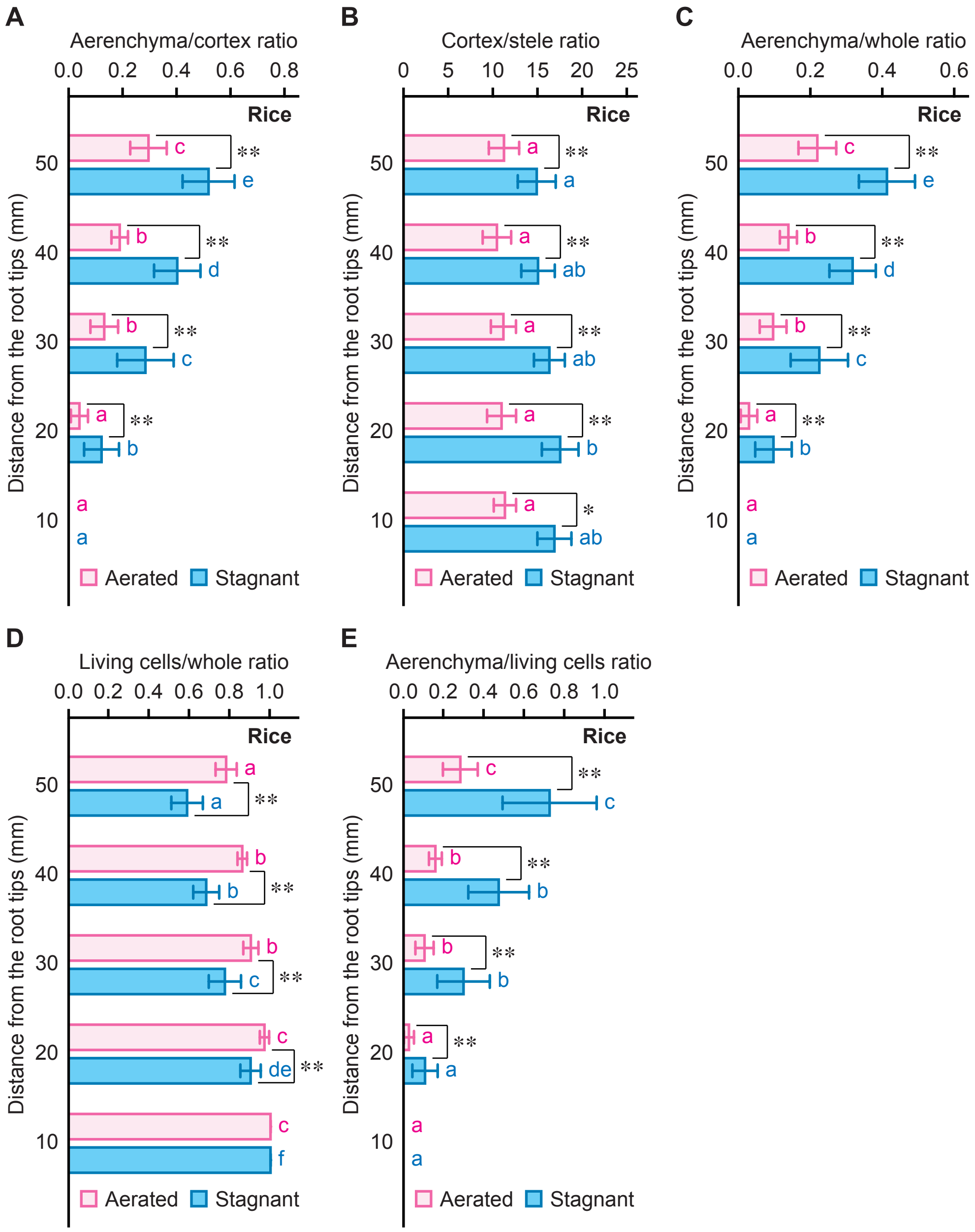

FIGURE S6 | Ratio of each tissue size in adventitious roots of rice. Ratio of aerenchyma to cortex **(A)**, cortex to stele **(B)**, aerenchyma to whole root **(C)**, living cells to whole root **(D)**, and aerenchyma to living cells **(E)** at 10, 20, 30, 40, and 50 mm from the tips of adventitious roots of rice under aerated or stagnant conditions. Nine-day-old rice seedlings were further grown under aerated or stagnant conditions for 7 days. Significant differences between under aerated and stagnant conditions at $P <$ 0.01 (two-sample t test) are denoted by **. Different lower-case letters denote significant differences among different positions of roots ($P < 0.05$, one-way ANOVA and then Tukey' s test for multiple comparisons). Values are means ± SD (n = 9).

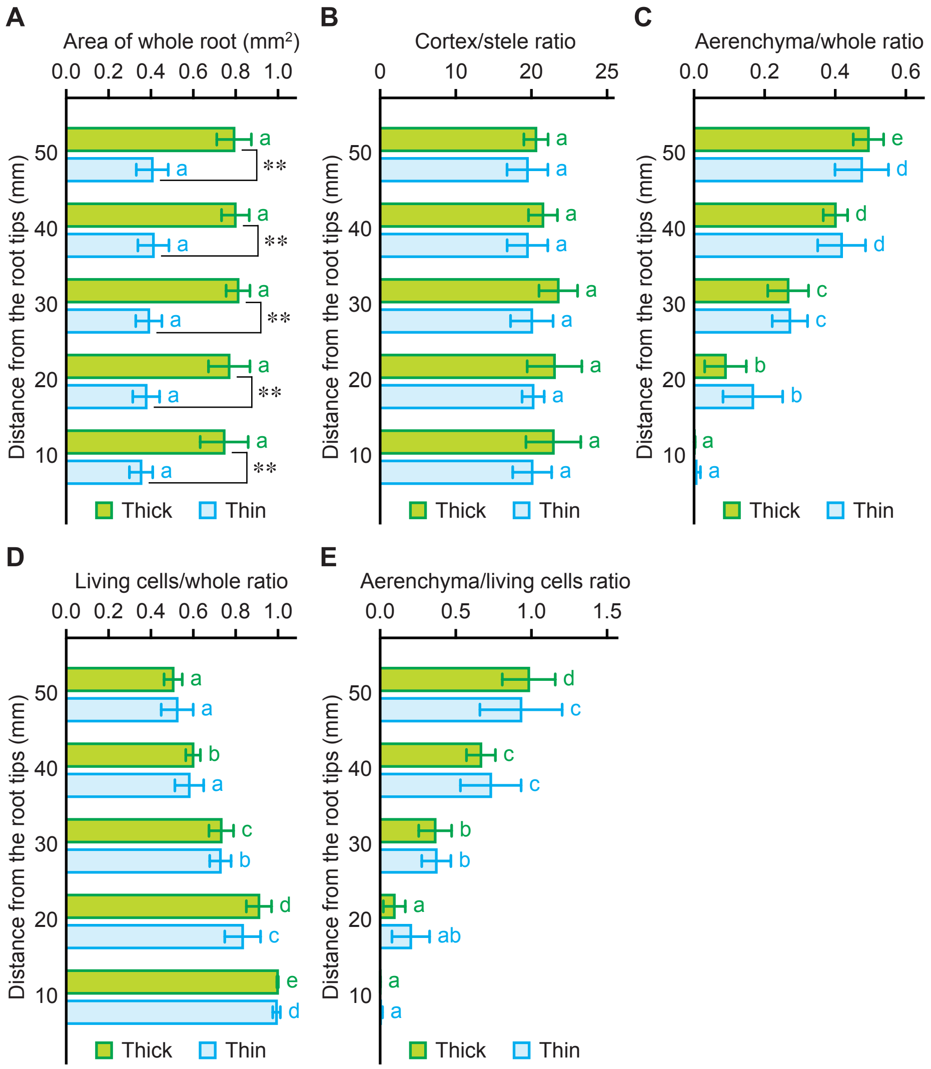

FIGURE S7 | Anatomy of thick and thin adventitious roots of rice. Area of whole root **(A)**, and ratio of cortex to stele **(B)**, aerenchyma to whole root **(C)**, living cells to whole root **(D)**, and aerenchyma to living cells **(E)** at 10, 20, 30, 40, and 50 mm from the tips of thick and thin adventitious roots of rice seedlings under stagnant conditions. Nine-day-old rice seedlings were further grown under stagnant conditions for 14 days. Significant differences between thick and thin roots at $P < 0.01$ (two-sample t test) are denoted by **. Different lower-case letters denote significant differences among different positions and types of roots ($P < 0.05$, one-way ANOVA and then Tukey' s test for multiple comparisons). Values are means ± SD (n = 8).

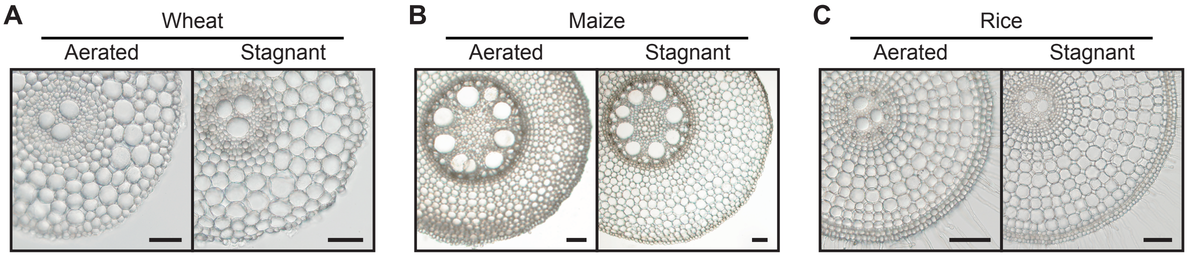

FIGURE S8 | Transverse sections at 10 mm from the root tips of adventitious roots of wheat, maize, and rice. Cross sections at 10 mm from the tips of adventitious roots of wheat **(A)**, maize **(B)**, and rice **(C)** under aerated or stagnant conditions. Nine-day-old wheat, maize, and rice seedlings were further grown under aerated or stagnant conditions for 7 days. Bars = 100 μm.
